# Maximizing catalytic efficiency with bio-inspired hierarchical porous zeolites

**DOI:** 10.1093/nsr/nwad155

**Published:** 2023-05-25

**Authors:** Svetlana Mintova

**Affiliations:** Laboratory of Catalysis and Spectrochemistry (LCS), ENSICAEN, CNRS, Normandy University, France

Hierarchical porous materials are characterized by their elegant interconnectivity and regularity across multiple length scales, which enables rapid mass transfer, exchange, and high catalytic performance [1]. These advanced materials differ from single-length-scale porous materials like microporous zeolites [2], mesoporous MCM-41 [3], SBA-15 [4] and MOF [5], and they are considered to be the second generation of porous materials [1,6]. These materials have attracted significant academic and industrial attention [7].

Su's group has made outstanding contributions to the development of hierarchical porous materials over the past 20 years [1,6,8]. Despite these significant advances, it is still a great challenge to achieve optimized materials with a defined porous hierarchy to maximize their performance due to a lack of design strategy. Su's group has taken another step forward in establishing the design principles of hierarchical porous materials [9]. However, introducing interconnected mesopores and macropores into single microporous zeolite crystals with the desired pore size at each length level remains highly challenging.

**Figure 1.**
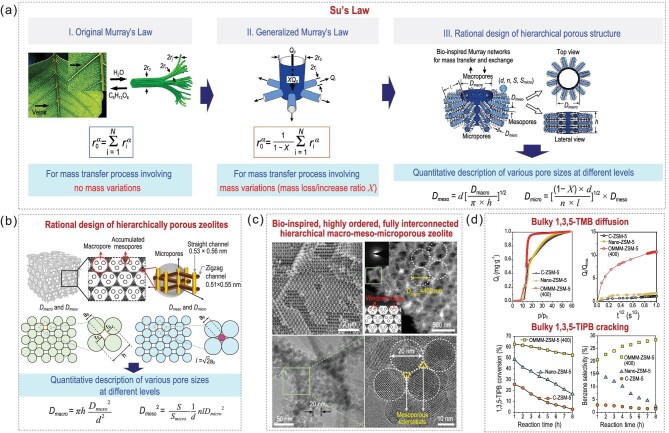
(a and b) Theoretical design. Parameters in a-I and II: *r_0_*, parent circular pore; *r_i_*, children pores; *α*, 2 or 3 (liquid phase: 2 and gaseous phase: 3); *N*, number of branches. Parameters in a-III: *D*, pore diameter; *n*, micropore amounts; *l*, mesopore length; *d*, size of nanoparticles; *X*, ratio of mass variation [9]. Parameters in b: *h*, macropore length; *l*, mesopore distance; *h*, macropore distance; *S*, BET surface area; *S_micro_*, micropore surface area [9]. (c) Targeted construction [11] and (d) performance evaluation [11] of bio-inspired hierarchical porous zeolites. Adapted with permission from Refs [9,11]. Copyright 2022 Oxford Academic and 2017 Springer Nature.

Nature has created various hierarchical porous living organisms that optimize mass transport and minimize energy consumption. These organisms can provide important inspiration for identifying rules to follow and emulate. Su's group recently revisited Murray's Law [10] by considering mass variation and constant surface substance exchange during mass transportation (Fig. 1a I–III) and used it as a material design principle to achieve the first bio-inspired materials with full interconnectivity of pores. In these highly ordered, bio-inspired hierarchical porous materials, the pore sizes decreased across multiple scales and finally terminated in a size-invariant unit resembling the hierarchical structure of leaf veins, vascular and respiratory systems. These biomimics effectively accelerated mass transfer and enhanced catalytic, sensing and electrochemical performance in liquid-solid, gas-solid and electrochemical reactions. This is the first example of realizing ‘material properties by design.’ The complex physical structure of hierarchically porous materials can now be expressed by a mathematical law [9].

This groundbreaking work has also inspired the quantitative design and targeted synthesis of high-performance hierarchical porous zeolites (Fig. 1a and b). In their recent work, Su's Law was extended to establish the relationship between the structural hierarchy, diffusion property, and catalytic performance in zeolite catalysis [11]. The resulting zeolite catalyst had a fully interconnected and highly ordered macro-meso-microporous structure with a pore-size ratio at each length scale designed rationally (Fig. 1c). This catalyst exhibited accelerated mass transfer with a relative diffusion rate of bulky 1,3,5-trimethylbenzene nine times higher and improved catalytic performance with 1,3,5-triisopropylbenzene cracking TOF ten times higher than their microporous counterparts (Fig. 1d). This work reports the first targeted synthesis of hierarchical porous zeolites following design rules that have evolved in natural hierarchical systems.

The pioneering studies conducted by Su's team demonstrate that bio-inspired hierarchically porous materials, exhibit exceptional performance [12]. It is envisaged that such material design principles are versatile and can be applied to a wide range of porous materials, enabling the rational design and synthesis of bio-inspired hierarchical porous materials with targeted structures to optimize their properties.
